# The effects of competition and bundled payment on patient reported outcome measures after hip replacement surgery

**DOI:** 10.1186/s12913-021-06397-1

**Published:** 2021-04-26

**Authors:** Fanny Goude, Sverre A. C. Kittelsen, Henrik Malchau, Maziar Mohaddes, Clas Rehnberg

**Affiliations:** 1 Department of Learning, Informatics, Management and Ethics, Karolinska Institutet, Tomtebodavägen 18A, 17177 Stockholm, Sweden; 2 Centre for Health Economics, Informatics and Health Services Research, Stockholm Health Care Services, Region Stockholm, Tomtebodavägen 18A, 17177 Stockholm, Sweden; 3 Frisch Centre, Gaustadalléen 21, 0349 Oslo, Norway; 4 Department of Orthopaedics, Massachusetts General Hospital, Harvard Medical School, 55 Fruit Street, Boston, MA 02114 USA; 5 Department of Orthopaedics, Institute of Clinical Sciences, Sahlgrenska Academy, Gothenburg university, Medicinaregatan 3, 41390 Göteborg, Sweden; 6 Swedish Hip Arthroplasty Register, Centre of Registers Västra Götaland, Medicinaregatan 18 G, 41345 Göteborg, Sweden; 7 Department of Orthopaedics, Sahlgrenska University Hospital, Göteborgsvägen 31, 431 80 Mölndal, Sweden

**Keywords:** Patient reported outcome measures, Competition, Bundled payment, Quality, Difference-in-difference analysis, Total hip replacement, Entropy balancing, Patient choice, Economic incentives, Propensity scores

## Abstract

**Background:**

Competition-promoting reforms and economic incentives are increasingly being introduced worldwide to improve the performance of healthcare delivery. This study considers such a reform which was initiated in 2009 for elective hip replacement surgery in Stockholm, Sweden. The reform involved patient choice of provider, free establishment of new providers and a bundled payment model. The study aimed to examine its effects on hip replacement surgery quality as captured by patient reported outcome measures (PROMs) of health gain (as indicated by the EQ-5D index and a visual analogue scale (VAS)), pain reduction (VAS) and patient satisfaction (VAS) one and six years after the surgery.

**Methods:**

Using patient-level data collected from multiple national registers, we applied a quasi-experimental research design. Data were collected for elective primary total hip replacements that were carried out between 2008 and 2012, and contain information on patient demography, the surgery and PROMs at baseline and at one- and six-years follow-up. In total, 36,627 observations were included in the analysis. First, entropy balancing was applied in order to reduce differences in observable characteristics between treatment groups. Second, difference-in-difference analyses were conducted to eliminate unobserved time-invariant differences between treatment groups and to estimate the causal treatment effects.

**Results:**

The entropy balancing was successful in creating balance in all covariates between treatment groups. No significant effects of the reform were found on any of the included PROMs at one- and six-years follow-up. The sensitivity analyses showed that the results were robust.

**Conclusions:**

Competition and bundled payment had no effects on the quality of hip replacement surgery as captured by post-surgery PROMs of health gain, pain reduction and patient satisfaction. The study provides important insights to the limited knowledge on the effects of competition and economic incentives on PROMs.

**Supplementary Information:**

The online version contains supplementary material available at 10.1186/s12913-021-06397-1.

## Background

Market-based healthcare reforms are increasingly being implemented worldwide to improve the performance of healthcare delivery [1–3]. For example, patient choice policies promoting fixed-price competition among providers have been a popular approach in the Northern European countries where arguments for choice have been increased efficiency, responsiveness to quality and flexibility, as well as patient empowerment [3]. Previous literature on the impact of competition on the performance have mainly focused on length of stay and failure-based indicators, such as mortality, readmission and complication rates, and shows mixed evidence on the effects [4–7].

Provider payment models have furthermore been reformed to reward efficiency and quality in many OECD-countries. Examples of such innovations include pay-for-performance schemes and bundled payment arrangements where providers are given economic incentives to improve their performance [8]. The effects of various payment models on the performance have previously been investigated and findings from these studies are inconclusive [9–11].

Patient reported outcome measures (PROMs) have gained an increasingly important role in policy evaluations and performance assessments [12]. From a patient’s perspective, PROMs can offer valuable information to decision-makers, healthcare providers and patients in terms of a quantified response (health gain) to a treatment. However, the literature on the effects of competition and economic incentives on PROMs is limited. A few recent studies have investigated the competition-induced reforms to the English National Health Service, where Skellern [13] found negative effects on PROMs of health gains for hip and knee replacement patients, whereas Feng et al. [14] found no association between increased competition and patient-reported health gains for hip replacements. Furthermore, the effects of economic incentives on PROMs have been analyzed in a study considering a value-based reimbursement program for spine surgery in Sweden, though no effects were found [15]. Lastly, a recent study found that patients at hospitals participating in Medicare’s bundled payment programs do not have meaningfully worse improvements in PROMs after hip or knee replacement as patients at non-participating hospitals [16].

We contribute to this limited knowledge by evaluating the impact of a competition-induced reform with economic incentives for elective hip replacement surgery on PROMs in Region Stockholm, Sweden. The reform led to patient choice of provider, free entry of new providers through accreditation, and a bundled payment model being implemented. By introducing competition on the market and giving the providers economic incentives, the reform primarily aimed to shorten waiting times which were unacceptably long at the time of implementation, as well as to empower the patient and improve provider quality and efficiency [17]. In a previous study, we found that this reform increased the length of stay in conjunction with the surgical admission, reduced complication rates within 90 days following the surgery and had no effects on patient satisfaction with the surgical outcome 1 y after surgery [6]. Moreover, a report by Wohlin et al. [18] indicate that the same reform was associated with reductions in waiting times, resource use and complication rates within 2 y after surgery, but not associated with patient reported pain reduction and health-related quality of life (EQ-5D). However, in most of their analyses, causality was not captured. The present study aims to examine the effects of competition and bundled payment on the perceived quality of elective hip replacement surgery as captured by PROMs of health gain, pain reduction and patient satisfaction one and 6 y after the surgery.

## Methods

### Setting

The Swedish healthcare system is mainly tax-funded and decentralized, where the 21 regions are responsible for healthcare funding and delivery. In recent years, more than 18,000 primary hip replacement surgeries are performed each year at around 75 different orthopaedic providers (mainly region-owned local, central and university hospitals, but also some private specialized centres) [19]. The providers are in general reimbursed through the Diagnosis Related Group (DRG)-model, either as budget or as activity-based funding.

In January 2009, Region Stockholm introduced a reform for elective total hip and knee replacement surgery which led to patient choice of provider, free entry of new providers through accreditation, and a bundled payment model being implemented. The reform is limited to low-risk profile patients (patients with American Society of Anaesthesiologists (ASA) grade 1–2), who can freely choose between several authorized providers [17]. High-risk profile patients are mainly referred to central and university hospitals. Before the reform, patients were only entitled to choose provider within primary and outpatient care. Patients were (and still are) furthermore covered by the national healthcare guarantee, meaning that if the waiting time for treatment (including inpatient care) was exceeded, patients were offered care elsewhere, although without possibility to choose where.

In order for providers to be accredited, certain criteria have to be met, including requirements for reporting data on quality indicators and a minimum of 50 surgeries per year for the operating surgeon. Furthermore, the providers are not limited in production volume [17]. In 2009, all emergency hospitals (six region-owned and one privately-owned) and three private specialized centres in Stockholm applied to become authorized care providers, in addition to one new private specialized centre. During the remaining study period, no provider entered or left the market.

The reimbursement scheme in Stockholm changed from a DRG-based arrangement to a bundled payment model for this patient group. With this model, providers are given a lump sum payment per patient to cover costs for a defined care chain, including pre-operative diagnostics, surgery, post-operative care and complications within 2 y after the surgery. As part of the bundled payment model, a performance-payment of a few percentages is further used where the providers are compensated for reaching certain performance targets. These targets include the proportion of patients who experience improved quality of life and pain relief 1 y after surgery. The region collects data on performance indicators from the local patient administrative system and the national quality registries to monitor the providers [17].

### Data

The Swedish Hip Arthroplasty Register (SHAR) was used to identify elective primary total hip replacements due to osteoarthritis. Data were collected for surgeries that were carried out between 2008 and 2012, and contain information on patient demography, the surgery and PROMs at baseline and at one- and six-years follow-up. To determine the comorbidity of the patients, previous use of hospital inpatient care within 1 y prior to surgery were collected from the national Patient Register. Furthermore, data on patients’ level of education and civil status were collected from the Swedish Longitudinal Integrated Database for Health Insurance and Labour Market Studies. The data from these two registers were linked to SHAR through personal identification numbers and the combined dataset was subsequently anonymized.

**Fig. 1:**
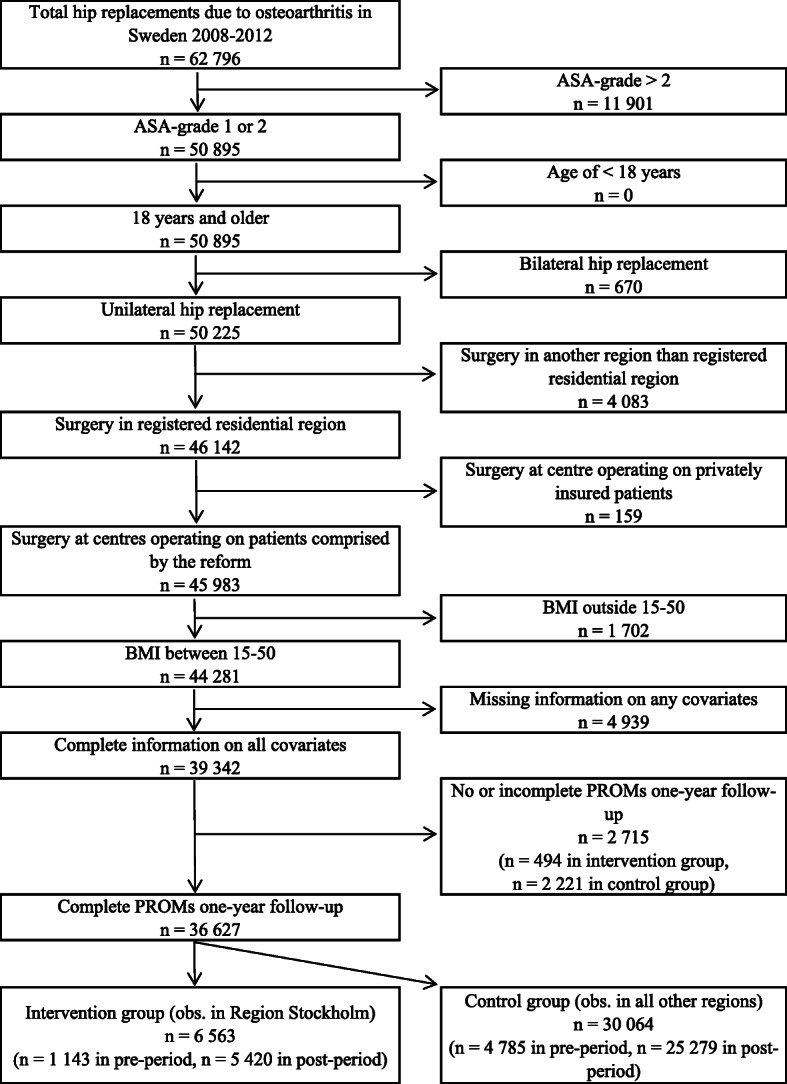
Flowchart, one-year follow-up. Flowchart of the study. ASA, American Society of Anaesthesiologists; obs., observations; PROMs, patient reported outcomes measures

We included patients covered by the reform, i.e., patients with ASA-grade 1 or 2. We excluded patients below 18 years of age, patients with a BMI outside the range of 15–50 and patients with missing information on any of the PROMs (at baseline or at follow-up) or covariates. Patients who underwent bilateral hip replacement or underwent surgery in another region than their registered residential region were further excluded. In addition, we excluded all patients at a private specialized centre in Stockholm which mainly performs surgery on privately insured patients who are not affected by the reform. The intervention group was defined as all patients in Stockholm, whereas the control group was defined as all other patients from the other regions in Sweden. The number of observations meeting the above inclusion and exclusion criteria for the one-year follow-up are illustrated in Fig. 1 (see Supplementary Fig. 1 for the six-years follow-up).

### Outcome measures

SHAR, established in 1979, has been collecting patient-level data from all orthopaedic departments performing total hip replacements in Sweden since over 40 years, which makes it one of the oldest national quality registers in the country. The register has been collecting PROMs since 2007. Prior to the surgery, patients are asked to respond voluntarily to a questionnaire covering the generic EQ-5D survey of health-related quality of life [20] and a visual analogue scale (VAS) ranging from 0 to 100 for current pain level. The EQ-5D survey consists of two components; a VAS ranging from 0 to 100 for current health status estimation and the EQ-5D index which captures information on current health status in five dimensions (mobility, self-care, usual activities, pain/discomfort, and anxiety/depression). When SHAR first started collecting this information, a three-level scale was used for each dimension (having no, some or extreme problems). Using weights obtained from population-level surveys [21], the response profile is then converted into a single-dimensional measure of health-related quality of life, referred to as the EQ-5D index. The index ranges from 1 (for no problems on all dimensions, i.e., the best health state) to − 0.594 (for the worst health state). The patient receives another questionnaire one, six and 10 y after the surgery covering the same PROM items as well as a supplementary VAS (ranging from 0 to 100) for satisfaction with the outcome of the surgery. The ideal post-operative state of the patients is 1 for EQ-5D index, and 100 for health status and 0 for pain score as well as satisfaction on the VAS. The compliance in the PROMs collection has been around 90% on a national level.

In this study, we were interested in the effects of the reform on health gain and pain reduction after hip replacement surgery. Health gain (pain reduction) is defined as the change from pre-surgery health (pain) status to post-surgery health (pain) status. Our outcome measures were gain in EQ-5D index (*EQ-5D_index*), gain in health status according to VAS (*Health_VAS*) as well as reduction in pain level according to VAS (*Pain_VAS*) one and 6 y after the surgery. Furthermore, one- and six-years post-surgery satisfaction with the outcome of the surgery according to VAS (*Satisfaction_VAS*) were also used as outcome measures.

### Statistical analyses

The study aimed to assess the causal treatment effect of the reform on the selected PROMs. However, when analysing observational data, there is a potential for selection bias since without randomization, the treatment and control groups could be different in ways that affect the outcomes. With inspiration from Achelrod et al. [22] and Stuart et al. [23], we used difference-in-difference (DiD) analyses in conjunction with entropy balancing in order to reduce confounding arising from selection bias.

DiD analysis is a quasi-experimental research design which compares changes in an outcome between treatment groups before and after an intervention. With this method, unobserved time-invariant differences between treatment groups are accounted for. The approach has often been applied to evaluate the effects of healthcare interventions and policies [24–28]. However, one concern using the DiD method, especially when data comes from repeated cross-sections as in this study, is that the composition of patients in the treatment groups may be time-varying or vary in ways that would affect their trends. To account for this type of time-varying confounding [23, 29], entropy balancing can be useful. Entropy balancing is a data preprocessing technique which uses a reweighting scheme to create unit weights so that the covariate distributions in the reweighted treatment groups satisfy a set of prespecified balance constraints. In the process, differences in the distributions with respect to the first, second, or higher moments are exactly adjusted for [30, 31]. In this study, we applied an entropy balancing algorithm to achieve balance in the mean and variance for a set of covariates between the treatment groups.

The data were collapsed into two periods, pre-reform (2008) and post-reform (2009–2012). Thereafter, following Stuart et al. [23] and Blundell and Dias [32], the entropy balancing algorithm was applied to find weights for the patients in Stockholm before the reform, and for the patients in the control group before and after the reform to make them all comparable to the patients in Stockholm after the reform. Included covariates were age, sex, ASA-grade, BMI, Charnley classification (a patient self-reported comorbidity grouping for walking ability), level of comorbidity as indicated by Elixhauser Comorbidity Index, surgical approach, educational level, civil status as well as the pre-operative value of the respective PROM (except for satisfaction, which is measured only post-operatively). The weights were estimated separately for each outcome measure and follow-up period (one and 6 y). The balance of covariates was assessed by comparing weighted means and variances.

The DiD analyses, used to estimate the causal treatment effects, were performed using weighted regression modelling with the weights produced by the entropy balancing algorithm to ensure balance on the covariates. In addition, the same set of covariates from the balancing algorithm were adjusted for in the DiD analyses to better isolate the treatment effect. As the data were aggregated into two periods (before and after the reform), problems of serially correlated outcomes were avoided [33]. The standard errors were adjusted for clustering of patients within hospitals. All statistical analyses were conducted using the SAS software, version 9.4 [34]. The SAS-codes provided by Faries et al. [31] were used for the entropy balancing.

### Sensitivity analyses

To test the robustness of the results, we first combined the DiD analyses with propensity scores. Originally designed to correct for different propensities to be treated by modelling the selection process as a function of the covariates [35], propensity scores have been commonly used also to control for differences between control and treatment groups when the selection process is known, as is the case here.

Propensity scores represent the conditional probability of being in the treatment group given a set of covariates [31, 35, 36]. The propensity scores were computed using logistic regression models, adjusted for the previously mentioned covariates. The produced propensity scores were thereafter used in an inverse probability of treatment weighing scheme to provide weights for balance of the covariates. As in the main analysis, the weighing process was implemented three times for each outcome measure and follow-up period. Following Austin [35, 37, 38] and Faries et al. [31], balance was assessed through standardized mean differences and variance ratios, adjusted for the individual inverse propensity weights. The weights were then incorporated in the DiD regression models.

Most of the previous studies combining DiD analyses with methods to minimize selection bias have dealt with confounding arising across groups, however, they are limited in dealing with time-varying confounding [23, 29]. Nevertheless, with repeated cross-sections, the time dimension may not be necessary in situations where the levels of imbalance are low [39]. In the absence of longitudinal data, DiD models require that similar groups are observed at different times so that outcome differences between periods would have been parallel in the control and treated groups had the latter not been treated [32]. In a second sensitivity analysis, we therefore overlooked the time dimension to test whether this changed the results. This means that we only applied entropy balancing at each time period to deal with confounding arising across groups. I.e., weights were retrieved so that patients in the pre-period control group were comparable to patients in Stockholm in the pre-period, and similarly, so that patients in the post-period control group were comparable to the patients in Stockholm in the post-period. The weights were estimated separately per outcome measure and follow-up period.

## Results

For the one-year follow-up PROMs, a total of 36,627 observations were included in the analysis (Fig. 1), of which 6563 were from Stockholm (1143 and 5420 observations pre respectively post the reform) and 30,064 were from the other regions (4785 and 25,279 observations pre respectively post the reform). For the six-years follow-up PROMs, a total of 18,145 observations were included (Supplementary Fig. 1).

Descriptive statistics of the treatment groups prior to and post entropy balancing are presented in Table 1 for the one-year follow-up, and in Supplementary Table 1 for the six-year follow-up. Prior to entropy balancing, we observe that age, ASA-grade, BMI, distribution of Charnley classification and baseline values of PROMs were similar across the treatment groups and across time. Stockholm had a lower share of male patients, married patients, patients with low educational level and patients with comorbidities. Furthermore, it was more common with a direct lateral surgical approach in Stockholm. Table 1 and Supplementary Table 1 confirms that the entropy balancing was successful in creating balance in all covariates.

**Table 1:** Baseline characteristics of treatment groups prior to and post entropy balancing, one-year follow-up

	2008								2009–2012					
	Stockholm				Control				Stockholm		Control			
	*n* = 1143				*n* = 4785				*n* = 5420		*n* = 25,279			
	Before EB		After EB		Before EB		After EB				Before EB		After EB	
Covariates	Mean	Variance	Mean	Variance	Mean	Variance	Mean	Variance	Mean	Variance	Mean	Variance	Mean	Variance
Age	68.02	93.66	67.46	93.07	68.15	90.94	67.46	93.07	67.46	93.07	68.32	89.34	67.46	93.07
Gender														
Female	0.66	0.23	0.62	0.23	0.57	0.25	0.62	0.23	0.62	0.23	0.57	0.24	0.62	0.23
Male	0.34	0.23	0.38	0.23	0.43	0.25	0.38	0.23	0.38	0.23	0.43	0.24	0.38	0.23
ASA-grade														
1	0.31	0.21	0.29	0.21	0.30	0.21	0.29	0.21	0.29	0.21	0.29	0.21	0.29	0.21
2	0.69	0.21	0.71	0.21	0.70	0.21	0.71	0.21	0.71	0.21	0.71	0.21	0.71	0.21
BMI	26.32	15.18	26.30	14.93	27.12	17.75	26.30	14.93	26.30	14.93	27.26	17.47	26.30	14.93
Charnley Classification														
A	0.48	0.25	0.50	0.25	0.47	0.25	0.50	0.25	0.50	0.25	0.49	0.25	0.50	0.25
B	0.11	0.10	0.12	0.11	0.12	0.11	0.12	0.11	0.12	0.11	0.12	0.11	0.12	0.11
C	0.41	0.24	0.37	0.23	0.41	0.24	0.37	0.23	0.37	0.23	0.38	0.24	0.37	0.23
Elixhauser Index														
0	0.76	0.18	0.80	0.16	0.61	0.24	0.80	0.16	0.80	0.16	0.58	0.24	0.80	0.16
1	0.18	0.15	0.14	0.12	0.25	0.19	0.14	0.12	0.14	0.12	0.27	0.20	0.14	0.12
2	0.04	0.04	0.05	0.05	0.10	0.09	0.05	0.05	0.05	0.05	0.11	0.10	0.05	0.05
3	0.02	0.02	0.01	0.01	0.04	0.03	0.01	0.01	0.01	0.01	0.04	0.04	0.01	0.01
Surgical approach														
Posterior	0.27	0.20	0.20	0.16	0.58	0.24	0.20	0.16	0.20	0.16	0.60	0.24	0.20	0.16
Direct lateral	0.70	0.21	0.78	0.17	0.41	0.24	0.78	0.17	0.78	0.17	0.39	0.24	0.78	0.17
Other	0.03	0.03	0.01	0.01	0.01	0.01	0.01	0.01	0.01	0.01	0.01	0.01	0.01	0.01
Educational level														
Low	0.27	0.20	0.22	0.17	0.40	0.24	0.22	0.17	0.22	0.17	0.36	0.23	0.22	0.17
Middle	0.39	0.24	0.41	0.24	0.40	0.24	0.41	0.24	0.41	0.24	0.41	0.24	0.41	0.24
High	0.34	0.22	0.38	0.23	0.20	0.16	0.38	0.23	0.38	0.23	0.23	0.18	0.38	0.23
Civil status														
Unmarried	0.11	0.10	0.12	0.11	0.09	0.08	0.12	0.11	0.12	0.11	0.10	0.09	0.12	0.11
Married	0.53	0.25	0.53	0.25	0.60	0.24	0.53	0.25	0.53	0.25	0.59	0.24	0.53	0.25
Divorced	0.20	0.16	0.22	0.17	0.15	0.13	0.22	0.17	0.22	0.17	0.16	0.13	0.22	0.17
Widow/widower	0.15	0.13	0.13	0.11	0.16	0.13	0.13	0.11	0.13	0.11	0.15	0.13	0.13	0.11
PROMs baseline														
EQ-5D_index	0.44	0.10	0.45	0.10	0.43	0.10	0.45	0.10	0.45	0.10	0.44	0.09	0.45	0.10
Health_VAS	55.38	521.88	56.14	472.63	53.94	488.98	56.14	472.63	56.14	472.63	55.19	486.69	56.14	472.63
Pain_VAS	61.85	248.27	63.23	267.01	61.41	249.97	63.23	267.01	63.23	267.01	61.59	244.06	63.23	267.01
	**2008**								**2009–2012**					
	**Stockholm**				**Control**				**Stockholm**		**Control**			
**Outcomes**	**Mean**	**Variance**			**Mean**	**Variance**			**Mean**	**Variance**	**Mean**	**Variance**		
Gain EQ-5D_index	0.35	0.12			0.37	0.12			0.35	0.11	0.36	0.11		
Gain Health_VAS	21.90	766.63			23.36	718.07			21.82	671.09	22.44	684.77		
Reduction Pain_VAS	−48.86	504.02			−47.70	502.12			−49.94	510.58	−48.51	481.08		
Satisfaction_VAS	16.13	427.42			15.79	402.69			15.95	438.46	15.17	391.67		

Notes: *EB* entropy balancing

In the comparison of outcomes (Table 1 and Supplementary Table 1), we note that gains in EQ-5D index and health status according to VAS, reduction in pain as well as level of satisfaction after hip replacement surgery are similar across treatment groups. Furthermore, the outcomes before the reform are approximately the same after the reform, in both groups.

Results from the DiD analyses with weights from the entropy balancing are provided in Table 2 (for unweighted results, see Supplementary Table 2). All effect estimates are rather small, and none are statistically significant.

**Table 2:** Results from the DiD analyses based on entropy balancing

Outcomes	Unadjusted		Adjusted	
	DiD estimate	Std. Err.	DiD estimate	Std. Err.
One-year follow-up				
Gain EQ-5D_index	−0.004	0.017	−0.003	0.011
Gain Health_VAS	−0.202	1.129	−0.203	0.688
Reduction Pain_VAS	0.195	1.751	0.073	0.991
Satisfaction_VAS	0.320	1.296	0.310	1.366
Six-years follow-up				
Gain EQ-5D_index	0.011	0.015	0.012	0.010
Gain Health_VAS	1.317	1.575	1.238	1.455
Reduction Pain_VAS	0.715	1.747	0.650	0.974
Satisfaction_VAS	0.437	1.150	0.431	1.159

Notes: None of the estimates are statistically significant. *DiD* difference-in-difference, *Std. err.* standard error

### Sensitivity analyses

The inverse probability of treatment weighing scheme improved the balance between the treatment groups and across time (not shown here). All weighted standardized mean differences were less than 0.1 (recommended threshold), and the weighted variance rations were all between 0.5–2 (recommended threshold). Similarly, the entropy balancing which was performed separately at each time period was successful in creating balance in all covariates between the groups (not shown here).

The results from the DiD models in the sensitivity analyses are presented in Supplementary Table 3. The results are similar to those in the main analysis; small effect estimates, and none are statistically significant.

## Discussion

This study examined the effects of the simultaneous introduction of competition and a bundle payment model on PROMs after elective primary total hip replacement surgery in Stockholm. Using routine administrative data, we measured various PROMs: gain in health-related quality of life, pain relief and satisfaction one- and six-years post surgery. We combined entropy balancing with a DiD analytical framework and found that the reform did not have any significant effects on any of the included outcomes.

The post-surgery PROMs of health gain, pain reduction and patient satisfaction were at a relatively high level ex ante the reform in both groups, as shown in Table 1 and Supplementary Table 1, and the main driver behind these improvements is likely the fact of “having a new hip”. Moreover, health-related quality of life deteriorates with age and due to the bounded nature of PROMs, patients’ improvements in health are limited. Thus, it was not expected that the reform would have any major effects on the included outcomes. It could moreover be the case that the reform had heterogeneous effects on the different dimensions of the EQ-5D index. A future study could therefore decompose the results per dimension.

In view of the effects of this reform, the present results are in line with what was found regarding patient satisfaction in our previous study [6], as well as with what Wohlin and colleagues in general found regarding PROMs [18]. However, in our previous work, we also found that the reform successfully reduced complication rates within 90 days following surgery. It is reasonable to assume that complications are an indicator of health gains, i.e., as complication rates decrease, health gains are expected to increase. The extent of this, however, is likely to depend on the timing and type of complications. An explanation for this seemingly opposing finding may therefore be the timing discrepancy. At one- and six-years follow-up, patients have probably recovered from any complications occurring within 90 days after surgery, and if there had been any improvements in PROMs (because of fewer complications), these would have, most likely, appeared in the near future after the surgery.

Furthermore, in contrast to our study findings, Skellern [13] found that the competition-induced reform to the English National Health Service in 2006, in which patients requiring elective surgery were allowed to choose hospital was introduced, lowered care quality as captured by PROMs of health gain for hip and knee replacement patients. Reasons for this discrepancy may however be differences in the setting (e.g., the level of competition and design of economic incentives, and patient population) and methodology.

In their study on a similar competition-inducing reform with a bundle payment arrangement introduced in Stockholm, but for elective spine surgery, Eriksson and colleagues [15] also found no effects on PROMs. As they discuss in their paper, one reason for the lack of effect relates to the incentive structure. Within the spine surgery program, providers were given stronger financial incentives to avoid negative outcomes than to reach positive outcomes [15]. This is also the case for the reform being analyzed in the present paper. Within the bundled payment model, two types of outcomes with differences in the strength of incentives are considered. First, providers are responsible for covering healthcare costs related to the hip replacement surgery, including complications such as infection and revision surgery within 2 y post surgery. Second, the performance-payment, which is partly based on PROMs, is only a few percentages of the bundled payment in magnitude. Even if both outcomes are observable, providers are more strongly incentivized to avoid negative outcomes, e.g., complications, rather than to focus on PROMs. This may be supported by our previous findings of reduced complication rates [6], and could be another possible explanation for the lack of effects on PROMs.

### Limitations

The study is subject to a few limitations which should be noted. The first limitation concerns the design of the reform which combines a variety of features to improve the performance: increased competition from patient choice and encouragement of registration for new providers, and a bundled payment model. As these elements were introduced simultaneously, we are not able to separately examine each element and its effect on the outcomes.

Second, in order for the DiD estimates to be valid, the so-called common trends assumption must be fulfilled. Under this assumption, the outcomes for the treatment- and control groups follow the same trend before the reform and would have continued to follow the same trend in the absence of the reform. Since we only have data for 1 y prior to the introduction of the reform, we could not explore whether the groups followed the same trend. Nevertheless, we used weighing techniques to make the groups comparable to reduce selection bias arising from this type of time-varying confounding. There is also a possibility that other coincident policy initiatives or confounding events have affected the health gains differently in the different treatment groups and thus bias the results. However, we are not aware of such initiatives and events.

Third, the drawback of collapsing the data into a pre- and post-period is that information is lost and it is not possible to explore if, and how, the effects of the reform vary with time. A suggestion for future studies would therefore be to investigate this matter.

### Implications

According to economic theory, fixed-price competition and choice can drive quality improvements, which forms the basis for policy interventions. However, the success of this implication depends on several factors, such as the design of the payment system, the type of quality and whether patients take quality into account when making their choice of healthcare provider [40]. It has previously been found that patients undergoing elective hip replacement surgery do consider quality when making their choice of hospital [40–42]. Of particular interest is a study based on the English National Health Service, which showed that PROMs of health gains were more important in the choice of hospital than the more traditional quality measures [42]. Hence, publication of and access to quality information is an important factor for the outcome of fixed-price competition. In Sweden, SHAR publish annual reports containing various measures of provider quality (including PROMs), which are publicly available for patients. Yet, we know little about how, and on what basis, the choice of hospital is made by the patient alone, or in consultation with the referring physician. Thus, to gain a better understanding of the impact of the reform, further research is required to explore if, and how, patients are incorporating quality in determining hospital choice.

This study provides evidence that PROMs were left unaffected by the reform, which, however, need not be interpreted as a failure. Taken together with our previous finding of successfully reduced complication rates [6], it can rather be understood as the gains were either already good or appeared immediately after surgery. Alternatively, or in combination, the lack of effect relates to the incentive structure, where the principle and design of bundled payment focus on different quality measures. As mentioned, the incentive was mainly focused on avoiding negative outcomes, rather than improving PROMS. Given the high level of post-surgery PROMs of gains before the reform combined with weak incentives for improving positive outcomes, one could not expect such development. Such pre-conditions are important for policy makers to consider when financial incentives are designed and linked to outcomes within a payment model. One task is to review various quality indicators in terms of the magnitude of poor outcomes and the potential to achieve improvements. Quality indicators that are already at a satisfactory level should perhaps not become candidates for receiving additional rewards. By analyzing and identifying deficiencies in quality, payment models could be designed to better target relevant quality problems.

## Conclusions

By introducing competition on the market and giving the orthopaedic providers economic incentives through a bundled payment model, the reform aimed, among other things, to improve quality. PROMs can offer valuable information to decision-makers and healthcare providers and have gained an important role in policy evaluations and performance assessments. Considering hip replacement surgery quality as captured by post-surgery PROMs of health gain, pain reduction and patient satisfaction, we show that the reform had no effect on quality. To fully understand the underlying factors behind these results, further research is required. The study contributes to the limited knowledge on the effects of competition and economic incentives on PROMs.

## Supplementary Information

**Additional file 1: Supplementary Figure 1.** Flowchart, six-years follow-up.

**Additional file 2: Supplementary Table 1.** Baseline characteristics of treatment groups prior to and post entropy balancing, six-years follow-up.

**Additional file 3: Supplementary Table 2.** Results from the unweighted DiD analyses

**Additional file 4: Supplementary Table 3.** Results from the sensitivity analyses.

## Data Availability

The data that support the findings of this study are available from the Swedish Hip Arthroplasty Register, the National Board of Health and Welfare, and Statistics Sweden, but restrictions apply to the availability of these data, which were used under license for the current study, and so are not publicly available. Data are however available from the authors upon reasonable request and with permission of the Ethical Review Board, the Swedish Hip Arthroplasty Register, the National Board of Health and Welfare, and Statistics Sweden.

## Abbreviations

ASA: American Society of Anaesthesiologists
DiD: Difference-in-difference
DRG: Diagnosis Related Group
PROMs: Patient reported outcome measures
SHAR: The Swedish Hip Arthroplasty Register
VAS: Visual analogue scale
